# Network-Based Coexpression Analysis Identifies Functional and Prognostic Long Noncoding RNAs in Hepatocellular Carcinoma

**DOI:** 10.1155/2020/1371632

**Published:** 2020-10-06

**Authors:** Jianguo Li, Jin Zhou, Shuangshuang Kai, Can Wang, Daijun Wang, Jiying Jiang

**Affiliations:** Schools of Basic Medicine and Pharmacy, Weifang Medical University, 7166 Baotong West Street, Weifang, 261053 Shandong Province, China

## Abstract

Hepatocellular carcinoma (HCC) is a primary liver cancer associated with a growing incidence and extremely high mortality. However, the pathogenic mechanism is still not fully understood. In the present study, we identified 1,631 upregulated and 1,515 downregulated genes and found that cell cycle and metabolism-related pathways or biological processes highly dysregulated in HCC. To assess the biological importance of these DEGs, we carried out weighted gene coexpression network analysis (WGCNA) to identify the functional modules potentially involved in HCC pathogenesis or progression. The five modules were detected with Dynamic Tree Cut algorithm, and GO enrichment analysis revealed that these modules exhibited different biological processes or signaling pathways, such as metabolism-related pathways, cell proliferation-related pathways, and molecules in tumor microenvironment. Moreover, we also observed two immune cells, namely, cytotoxic cells and macrophage enriched in modules grey and brown, respectively, while T helper cell-2 (Th2) was enriched in module turquoise. Among the WGCNA network, four hub long noncoding RNAs (lncRNAs) were identified to be associated with HCC prognostic outcomes, suggesting that coexpression network analysis could uncover lncRNAs with functional importance, which may be associated with prognostic outcomes of HCC patients. In summary, this study demonstrated that network-based analysis could identify some functional modules and some hub-lncRNAs, which may be critical for HCC pathogenesis or progression.

## 1. Introduction

Hepatocellular carcinoma (HCC) is a primary liver cancer associated with a growing incidence and extremely high mortality, whose confirmed etiologic factors include hepatitis B/C, alcohol use, nonalcoholic steatohepatitis, and obesity [1]. Also, cirrhosis is regarded as an important indicator in the screening and surveillance of HCC [2]. The rapid progression often leads to poor prognosis of HCC as most diagnoses are made at advanced disease stages [3].

With the advance in biotechnologies, genomic causes behind HCC have been gradually revealed. Genomic analyses of HCC have identified some recurrently mutated genes, such as TERT promoter, TP53, CTNNB1, and AXIN1 [4]. Previous studies about microRNAs (miRNAs) show that miRNAs are closely related to HCC tumorigenesis, development and metastasis [5, 6]. For example, miR-188-5p can inhibit the proliferation and metastasis of HCC by targeting FGF5 [7]. Moreover, long noncoding RNAs (lncRNAs), which are generally unable to encode proteins, are also involved in tumor formation, development, or metastasis. Overexpression of lncRNA HULC in liver cancer promotes HCC proliferation by downregulating tumor suppressor gene p18 [8].

With the development of the biomarkers of HCC, the therapeutics has been greatly improved. However, for patients with advanced stages, the traditional surgical resection and chemotherapy are inadequacy. Transplantation, genomic-based, and immune therapies now become the center of attention as they exhibit a very promising effect on those virally induced cancers like HCC, and immunotherapy regarding immune checkpoint inhibitors has been applied clinically in cancers such as melanoma and non-small-cell lung cancer [9]. Infiltrating immune cells play a critical role in the surveillance and immune response of various solid tumors and contribute greatly to the identification of immunotherapy targets [10]. Infiltrating immune cells mainly fall into two groups: lymphoid and myeloid [11]. Recent study stated that the degree of immune cell infiltration into HCC is associated with divergent immune cell types and correlated to prognosis [12]. In the present study, we attempt to identify some key genes, functional modules, and pathways for HCC tumorigenesis and progression using network-based algorithm. The immune cells infiltrated in HCC tissues were also evaluated, and some critical lncRNAs were identified by the coexpression network. In summary, this study improved our understanding of HCC tumorigenesis and provided some potential therapeutic targets for HCC.

## 2. Materials and Methods

### 2.1. Data Collection

We collected RNA sequencing data of 50 HCC and 50 paired nontumor tissues from the Sequence Read Archive (SRA, https://www.ncbi.nlm.nih.gov/sra) database [13] with an accession number SRP068976 [14]. The SRA files were preprocessed by fastq-dump with the option *--split-files*, which generated two paired fastq files.

### 2.2. Read Mapping and Gene Expression Quantification

The RNA sequencing reads were first mapped to UCSC hg19 human reference genome (http://www.genome.ucsc.edu/) using hisat2 [15], and the alignments in SAM file were then sorted by samtools. The gene expression was quantified by StringTie [16] and ballgown pipeline, with the gene annotation from GENCODE v19 [17].

### 2.3. Differential Expression Analysis

The read count-based expression was used to identify the differentially expressed genes (DEGs) by R/bioconductor DESeq2 package [18]. The stably expressed genes were firstly identified if FPKM (fragment per kilobase million) > 1 in more than 20% samples. The differentially expressed genes were identified with the thresholds of BH (Benjamini and Hochberg) adjusted *P* value < 0.05 and fold change > 2 or < 1/2 [19, 20].

### 2.4. Weighted Gene Coexpression Network Analysis (WGCNA)

WGCNA [21] was performed to identify potential functional modules. The soft threshold for scale-free network was determined based on the maximal *R*-square (power = 9). TOM similarity was used to evaluate the distance between each gene pair. Moreover, hierarchical clustering analysis with average method and dynamic method was used to build the cluster tree and classify the genes into modules, respectively. We finally identified 5 functional modules.

### 2.5. KEGG, GO, and Immune Cell-Based Overrepresentation Enrichment Analysis

The KEGG (Kyoto Encyclopedia of Genes and Genomes) [22], GO (Gene Ontology) [23, 24], and immune cell-based overrepresentation enrichment analysis were implemented in R with clusterProfiler package [25], which used overrepresentation enrichment analysis (ORA) to identify enriched KEGG pathways, GO terms, and immune cells. The gene markers for immune cells were extracted from the previously published study [26]. The threshold for these gene sets was *P* value < 0.05.

### 2.6. Cox Regression Proportional Hazard Model-Based Survival Analysis

Cox regression proportional hazard model was used to evaluate the differences of overall survival between patients with two conditions, which was implemented in R programming software *survival* package with *coxph* function. To visualize the overall survival for each group, we used Kaplan-Meier curves to estimate the survival probability.

## 3. Results

### 3.1. Identification of Differentially Expressed Genes in HCC Tumors and Healthy Tissues

**Figure 1:**
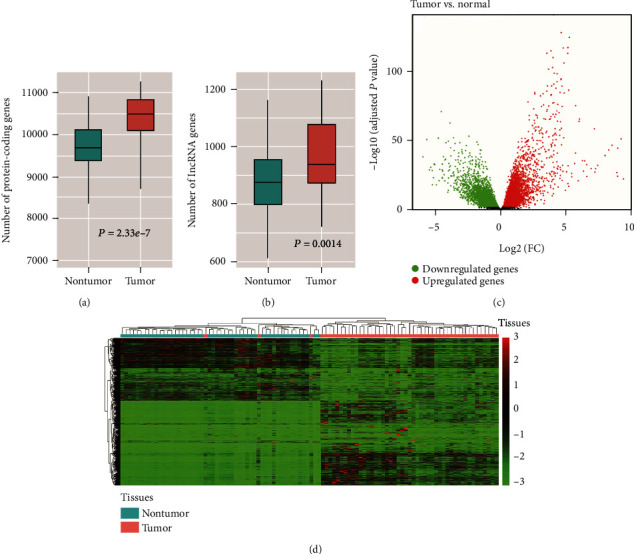
The overview of the protein-coding genes, lncRNAs, and differentially expressed genes. The distribution of number of protein-coding genes and lncRNAs was illustrated in (a) and (b). (c) The differentially expressed genes (DEGs) were represented by the points with colors red (upregulation) and green (downregulation). (d) The expression patterns of DEGs in tumor and nontumor tissues.

To uncover the dysregulated genes associated with HCC, we compared the gene expression profiles between tumor tissues and normal tissue adjacent to the tumor (NAT). From the HCC gene expression profiles, a total of 15,186 genes were identified (fragment per kilobase million, FPKM > 1 in more than 20% samples), while the number of protein coding genes (PCGs) and lncRNA genes significantly varied between tumor tissues and NAT, as more PCGs and lncRNAs were observed in tumor tissues compared with NAT (Wilcoxon rank-sum test, *P* < 0.05) (Figures 1(a) and 1(b)). Moreover, we observed quite dissimilar patterns regarding gene expressions between HCC tumor tissues and NAT (Figure 1(c), adjusted *P* value < 0.05 and log2 fold change > 1 or < −1), and identified 1,631 upregulated and 1,515 downregulated genes. Hierarchical clustering analysis was performed to further visualize expression patterns of the differentially expressed genes (DEGs), suggesting that there was a great difference between these two groups (Figure 1(d)).

### 3.2. Biological Interpretation of Differentially Expressed Gene Sets Utilizing GO and KEGG-Based Enrichment Analysis

**Figure 2:**
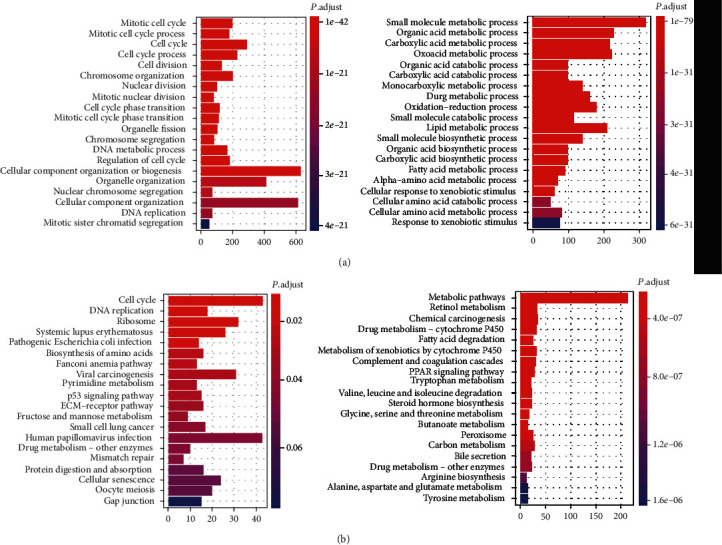
The GO biological processes and KEGG pathways enriched by the differentially expressed genes. (a) The GO biological processes enriched by the DEGs. The bars on the left and right represent the enriched GO terms enriched by the upregulated and downregulated genes, respectively. (b) The enriched KEGG pathways by the DEGs. The up- and downregulated genes were enriched in the pathways represented by the bars on the left and right, respectively.

To investigate the dysregulated signaling pathways and biological processes, we performed GSEA analysis of the DEGs based on using gene sets of KEGG and GO databases. KEGG-based enrichment analysis revealed that upregulated genes exhibited significant enrichment in pathways regarding cell division, cell replication, and other biological processes related to cell cycle, while downregulated genes were mainly involved in metabolic and catabolic processes (Figure 2(a)). GO enrichment analysis further confirmed our observations as upregulated genes were significantly enriched in cell cycle, DNA replication, and ribosome, while terms including metabolic pathways, fatty acid degradation, chemical carcinogenesis, and PPAR signaling pathway were significantly enriched of the downregulated genes (Figure 2(b)). Based on the KEGG and GO enrichment analysis, we observed that cell cycle and metabolism-related pathways or biological processes were up- or downregulated in HCC, suggesting that cell proliferation was hyperactivated and metabolic capability of liver was significantly decreased in HCC.

### 3.3. Coexpression Network Analysis of the DEGs

**Figure 3:**
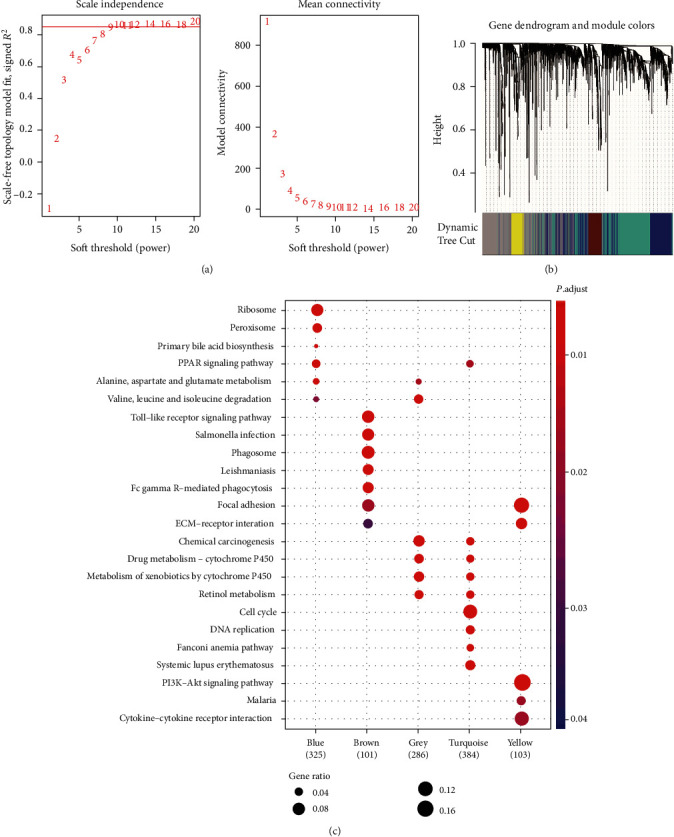
The weighted gene coexpression network analysis (WGCNA) of the DEGs. (a) The scale independence and mean connectivity used for the selection of soft power. (b) The hierarchical clustering analysis of the DEGs based on the TOM similarity. (c) The KEGG pathways enriched by the genes of WGCNA modules.

In order to assess the biological importance of these DEGs and the correlation patterns among them, weighted gene coexpression network analysis (WGCNA) was carried out. We chose soft power value 9 as it reflected the best scale independence and mean connectivity (Figure 3(a)). With this selected soft power, similarity matrices were calculated, and hierarchical clustering of these DEGs based on this dissimilarity measure was performed. Five modules were detected with Dynamic Tree Cut algorithm and distinguished by different colors (Figure 3(b)). After obtaining these module gene listings, the GO enrichment analysis was performed to interpret each module's biological functions. Except for a few shared terms, these modules exhibited little similarity in functions (Figure 3(c)). Genes in module 1 (blue, 325 genes in total) were mainly associated with ribosome, peroxisome, and PPAR signaling pathway, module 2 (brown, 101 genes in total) dealt with Toll-like receptor signaling pathway, salmonella infection, and phagosome, while module 5 (yellow, 103 genes in total) consisted of genes concerned with focal adhesion, ECM-receptor interaction, and PI3K-Akt signaling pathway. Modules 3 and 4 (denoted in grey and turquoise, including 286 and 384 genes, respectively) were both associated with retinol metabolism, metabolism of xenobiotics by cytochrome P450, drug metabolism-cytochrome P450, and chemical carcinogenesis. Specifically, module 2 (brown) was characterized by dysregulation of tumor microenvironment, such as ECM-receptor interaction and focal adhesion, while module 3 was enriched by the metabolism-related pathways. Moreover, we also found that cell proliferation-related pathways, such as cell cycle, DNA replication, and Fanconi anemia pathway, were enriched by the genes of module 4. Taken together, these modules were recognized as biologically meaningful in HCC patients.

### 3.4. Identification of Infiltrated Immune Cells in HCC Tissues

**Figure 4:**
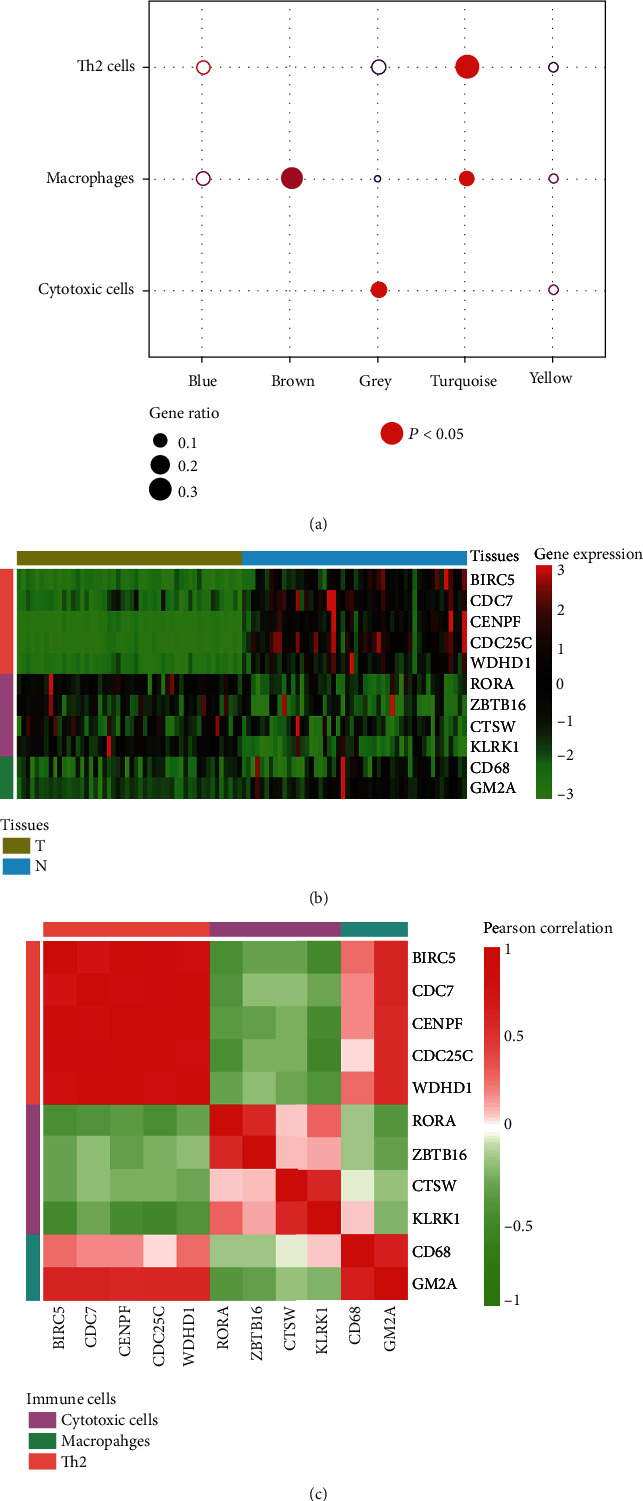
The immune cells aberrantly infiltrated in HCC tissues. (a) The three immune cell types enriched by the module genes. (b) The expression patterns of the immune cell-related marker genes in tumor and nontumor tissues. (c) The Pearson correlation coefficients between the markers within specific immune cell type or between the immune cells.

As the immune cells were infiltrated into the tumor tissues [27], we next investigated the infiltrated levels of the immune cells for the HCC tissues based on their marker gene sets. We performed gene set enrichment analysis (GSEA) to test the enrichment degree of the differentially expressed marker genes for each WGCNA module. Notably, two immune cells, namely, cytotoxic cells and macrophage, were enriched in modules grey and brown, respectively, while T helper cell-2 (Th2) was enriched in module turquoise (Figure 4(a)). Specifically, the marker genes of cytotoxic cells and macrophage were upregulated in HCC, and the marker genes of Th2 were downregulated in HCC, suggesting that cytotoxic cells and macrophage were highly infiltrated in HCC, while the Th2 cells were reduced in HCC tissues as compared with nontumor tissues. Moreover, the marker genes of the three immune cell types, including BIRC5, CDC7, CENPF, CDC25C, WDHD1, RORA, ZBTB16, CTSW, KLRK1, CD68, and GM2A, were observed to be dysregulated in HCC (Figure 4(b)). Correlation analysis revealed that marker genes within each immune cell were highly correlated with each other, suggesting that they could cooperate with each other to function in immune cell (Figure 4(c)). In addition, we also observed that the markers of cytotoxic cells and macrophages exhibited higher correlation, indicating that the two cell types may have interactions in HCC tissues.

### 3.5. Identification of Critical Hub-lncRNAs and Evaluation of Their Prognostic Power in HCC Patients

**Figure 5:**
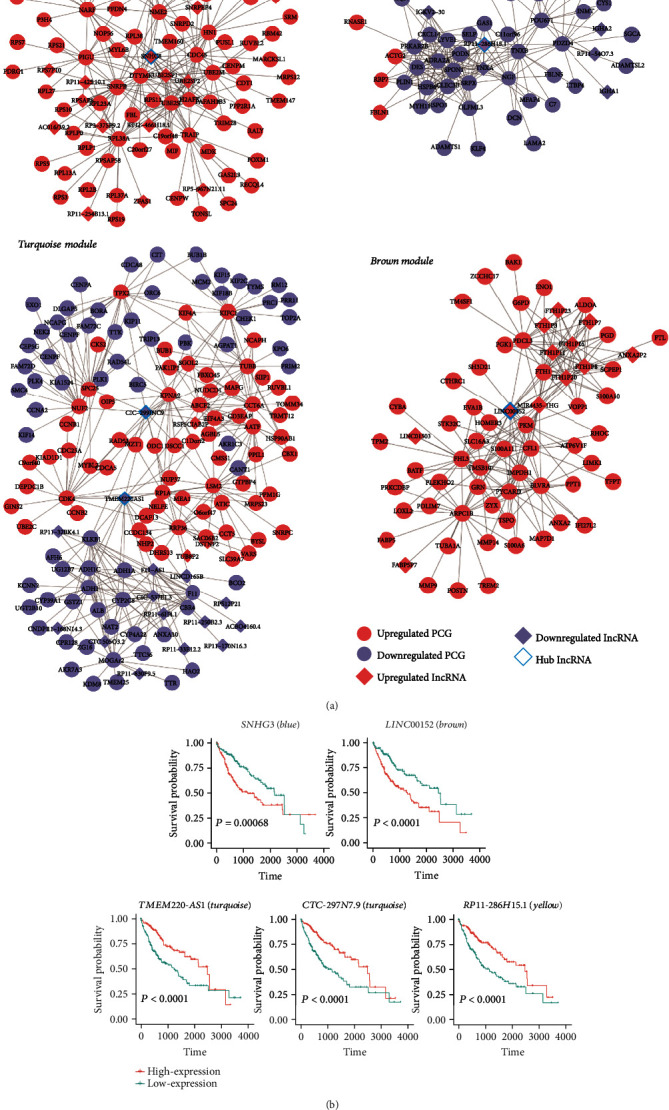
The hub-lncRNAs in WGCNA network and their prognostic association with HCC overall survival. (a) The visualization of the WGCNA modules with hub-lncRNAs by Cytoscape. (b) The KM curves of the hub-lncRNAs in TCGA-LIHC cohort.

To better summarize the functional roles of each module in HCC, it is important to recognize the intramodular interactions and representative genes in a coexpression network. Thus, using Cytoscape [28], we visualized the interaction networks of these genes based on their coexpression and uncovered hub-lncRNAs for each module, which may resemble functional importance (Figure 5(a)). We successfully identified SNHG3 in the blue module, LINC00152 in the brown module, TMEM220-AS1 and CTC-297N7.9 in the turquoise module, and RP11-286H15.1 in the yellow module as hub-lncRNAs. Notably, SNHG3 and LINC00152 were previously reported to function as competing endogenous RNA or regulate essential pathways to promote tumorigenesis [29, 30]. For each hub-lncRNA, samples were divided into high- and low-expression groups based on the expression of this hub-lncRNA. Utilizing the survival data of HCC patients in corresponding high- and low-expression groups from TCGA LIHC (liver hepatocellular carcinoma) datasets, Kaplan-Meier curves were plotted for each hub-lncRNA, and significant differences in overall survival were observed between high- and low-expression groups (*P* < 0.05, Figure 5(b)). These findings not only suggested that the identification of hub-lncRNA based on coexpression network could uncover lncRNAs with critical function but also revealed that these hub-lncRNAs had the power of evaluating prognostic outcomes in HCC patients.

## 4. Discussion

Hepatocellular carcinoma (HCC) is a primary liver cancer associated with a growing incidence and extremely high mortality. However, the pathogenic mechanism is still not fully understood. In the present study, we compared the gene expression profiles between tumor tissues and NATs and identified 1,631 upregulated and 1,515 downregulated genes. GSEA was subsequently performed to investigate the dysregulated signaling pathways and biological processes, which revealed that DEGs exhibited significant enrichment in cell cycle and metabolism-related pathways or biological processes. The hyperactivated cell cycle and metabolism-related pathways indicated that uncontrolled tumor cell proliferation and decreased metabolic capability may be the hallmark of HCC [31–33].

In order to assess the biological importance of these DEGs and the correlation patterns among them, weighted gene coexpression network analysis (WGCNA) was carried out. Five modules were detected with Dynamic Tree Cut algorithm (Figure 3(b)). After obtaining these module gene listings, the GO enrichment analysis was performed to interpret each module's biological functions. Except for a few shared terms, these modules exhibited little similarity in functions (Figure 3(c)). Specifically, the module 2 (brown) was characterized by dysregulation of tumor microenvironment, such as ECM-receptor interaction and focal adhesion, while module 3 was enriched by the metabolism-related pathways. Moreover, we also found that cell proliferation-related pathways, such as cell cycle, DNA replication, and Fanconi anemia pathway, were enriched by the genes of module 4. Taken together, these modules were recognized as biologically meaningful in HCC patients. In accordance with the characteristics of HCC subtypes [34–36], the pathways or biological processes characterized for the three modules may also be associated with the signatures of previous HCC subtypes. To further characterize the features of the WGCNA modules, we further tested the enrichment degree of the differentially expressed marker genes of immune cells for each WGCNA module. Notably, two immune cells, namely, cytotoxic cells and macrophage, were enriched in modules grey and brown, respectively, while T helper cell-2 (Th2) was enriched in module turquoise (Figure 4(a)). Even though cytotoxic cells and macrophages were highly infiltrated in HCC tissues, their immune activities were suppressed, indicating that the immune checkpoint inhibitors such as PD1/PDL1 and CTLA-4/B7-1/B7-2 may function in HCC tissues [37, 38]. Consistently, CTLA4 was highly expressed in HCC (*P* < 0.05).

Among the network constructed by WGCNA, SNHG3 in the blue module, LINC00152 in the brown module, TMEM220-AS1 and CTC-297N7.9 in the turquoise module, and RP11-286H15.1 in the yellow module were identified as hub-lncRNAs. SNHG3 and LINC00152 were previously reported to function as competing endogenous RNA or regulate essential pathways to promote tumorigenesis [29, 30]. Survival analysis of these hub-lncRNAs revealed that these hub-lncRNAs were closely associated with the HCC overall survival (*P* < 0.05, Figure 5(b)). These findings suggested that coexpression network analysis could uncover lncRNAs with functional importance, which may be associated with prognostic outcomes of HCC patients.

In addition, the present study also has some limitations. First, the relationship between suppressed activities of cytotoxic cells and macrophages and CTLA4 and the functional roles of the hub-lncRNAs should be further validated by experiments. Second, the prognostic values of the hub-lncRNAs should be validated in independent datasets. In summary, this study demonstrated that network-based analysis could identify some functional modules and some hub-lncRNAs, which may be critical for HCC pathogenesis or progression.

In summary, this study demonstrated that network-based analysis could identify some functional modules and some hub-lncRNAs, which may be critical for HCC pathogenesis or progression.

## Data Availability

We collected RNA sequencing data of 50 HCC and 50 paired nontumor tissues from Sequence Read Archive (SRA, https://www.ncbi.nlm.nih.gov/sra) database with an accession number SRP068976.
